# Uniformly Dispersed ZnFe_2_O_4_ Nanoparticles on Nitrogen-Modified Graphene for High-Performance Supercapacitor as Electrode

**DOI:** 10.1038/srep43116

**Published:** 2017-02-21

**Authors:** Lei Li, Huiting Bi, Shili Gai, Fei He, Peng Gao, Yunlu Dai, Xitian Zhang, Dan Yang, Milin Zhang, Piaoping Yang

**Affiliations:** 1 Key Laboratory of Superlight Materials and Surface Technology, Ministry of Education, College of Material Science and Chemical Engineering, Harbin Engineering University, Harbin 150001, P. R. China; 2 Key Laboratory for Photonic and Electronic Bandgap Materials, Ministry of Education, School of Physics and Electronic Engineering, Harbin 150001, P. R. China

## Abstract

A facile strategy has been adopted for the preparation of ZnFe_2_O_4_/NRG composite by anchoring ultrasmall ZnFe_2_O_4_ nanoparticles on nitrogen-doped reduced graphene (denoted as NRG) for high-performance supercapacitor electrode. Remarkably, the growth of ZnFe_2_O_4_ nanocrystals, the reduction of graphitic oxide and the doping of nitrogen to graphene have been simultaneously achieved in one process. It is found that the NRG employed as substrate can not only control the formation of nano-sized ZnFe_2_O_4_, but also guarantee the high dispersion without any agglomeration. Benefiting from this novel combination and construction, the hybrid material has large surface area which can provide high exposure of active sites for easy access of electrolyte and fast electron transport. When served as supercapacitor electrode, the ZnFe_2_O_4_/NRG composite exhibits a favorable specific capacitance of 244 F/g at 0.5 A/g within the potential range from −1 to 0 V, desirable rate stability (retain 131.5 F/g at 10 A/g) and an admirable cycling durability of 83.8% at a scan rate of 100 mV/s after 5000 cycles. When employed as symmetric supercapacitor, the device demonstrates favorable performance. These satisfactory properties of the ZnFe_2_O_4_/NRG composite can make it be of great promise in the supercapacitor application.

With the ever-increasing demand in the practical application of new-type portable electronics and electric vehicles, it is of great significance to develop energy storage devices with high performance and environmentally friendly properties^1^,^2^,^3^,^4^,^5^,^6^. Compared with the conventional dielectric capacitors and rechargeable batteries, supercapacitors have attracted more considerable attention owning to their high power density, long cycle lifetime and excellent reliability in the fields of energy storage and conversion^7^,^8^,^9^,^10^. In terms of the operational charge storage mechanism, supercapacitors can be classified into pseudocapacitors and electrochemical double layer capacitors (EDLCs)^11^,^12^,^13^,^14^,^15^. Especially, pseudocapacitors, based on reversible redox reactions between the electrolyte and the electrode bulk, can achieve admirable specific capacitance and energy density, which can be as promising candidate for constructing novel energy storage devices^16^,^17^,^18^,^19^. Accordingly, a variety of pseudocapacitive electrode materials, such as metal oxides/hydroxides and conductive polymer, have been employed to realize practical application of supercapacitor^20^,^21^,^22^,^23^. However, the performance of pseudocapacitors has often been restricted by low-electronic conductivity of the active materials resulting from insufficient transport for ion and electron at the electrode/electrolyte interface and in electrodes, leading to undesirable faradaic redox reactions in high current rate. To solve this problem, an effective strategy is to combine them with the highly conductive support materials, which can provide sufficient electroactive species exposed to electrolyte for the favorable reaction kinetics^24^,^25^.

Among various good conductive materials, graphene, as one of carbonaceous materials, represents an attractive substrate for the immobilization of active species to provide good electron transfer paths and improve stability of the entire hybrid, because of its porous two-dimensional structure, large specific surface area and exceptional high conductivity^26^,^27^,^28^. Simultaneously, it has been certified that graphene can act as a support to prevent the loaded nanomaterials from aggregation by balancing their high interface energy^29^,^30^,^31^. By the virtue of these advantages, a variety of electrode materials integrating pseudocapacitive component with graphene have been successfully constructed for achieving high-performance energy storage devices^32^,^33^,^34^. In addition, many investigations revealed that doping of heteroatoms (N and S) to the internal or surface carbon matrix, can significantly improve the performance of carbon-based supercapacitors and provide numerous active sites and extrinsic defects for the generation of pseudocapacitive nanomaterials^35^,^36^,^37^,^38^.

Recently, binary metal oxides (based on AB_2_O_4)_, especially spinel ferrites with the general formula MFe_2_O_4_ (M = Mn, Co, Ni, Zn or Mg), has been intensively studied as qualified pseudocapacitive electrode material^8^,^39^,^40^,^41^,^42^. Compared with the monometallic oxides, Fe-based binary oxides, triggering synergetic effect from both Fe and M ions, can offer richer redox chemistry to obtain higher specific capacitance^43^,^44^,^45^. As an important member of ferrite family, zinc ferrite (ZnFe_2_O_4_) exhibits promising potential for the application of supercapacitor, due to abundant resources, low cost, environmental friendliness and high electrochemical activity^46^,^47^. However, the ZnFe_2_O_4_ based electrode often suffers from low conductivity, leading to unsatisfactory rate capability^48^,^49^. Furthermore, the morphology and size of the electrode materials also play important roles in determining the performance of supercapacitors^50^,^51^,^52^,^53^,^54^,^55^. Therefore, it is significant to fabricate electrode material utilizing the combination of nano-sized ZnFe_2_O_4_ and nitrogen-doped graphene.

In this paper, we present the preparation of nano-sized ZnFe_2_O_4_ particles uniformly loaded on the surface of porous nitrogen-doped graphene (denoted as ZnFe_2_O_4_/NRG) without any agglomeration, *via* a facile solvothermal strategy followed by calcining treatment. It is worth noting that the solvent, N, N-dimethylmethanamide (DMF), plays dual important roles for the fabrication of NRG. On one hand, the DMF can provide nitrogen source for the chemical doping of graphene. On the other hand, GO can be reduced in the DMF solution which can be served as reducing agent under solvothermal condition and ensure the formation of ZnFe_2_O_4_ nanoparticles. With the doping of nitrogen element, the graphene can not only possess favorable structural stability at high rate, but also offer numerous active sites for the growth and bonding of ZnFe_2_O_4_ nanoparticles. This unique combination between ZnFe_2_O_4_ and NRG can remarkably enhance electrical conductivity and create a speedy diffusion way from the electrolyte to the electrode. When applied as supercapacitor electrode, the ZnFe_2_O_4_/NRG shows favorably electrical performance including specific capacitance, rate capability and cycling performance. When fabricated as symmetric device, the material also exhibits ideal electrochemical performance. These results demonstrate that this process towards graphene and binary metal oxides is promising for the future fabrication of supercapacitor electrode material.

## Results and Discussion

### Phase and morphology properties

**Figure 1:**
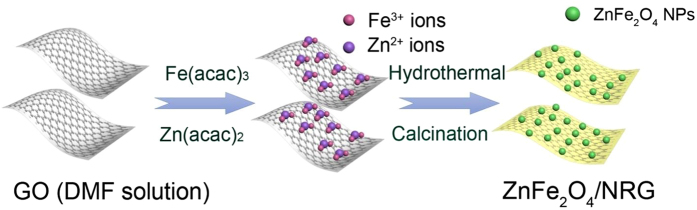
Schematic illustration for the preparation of ZnFe_2_O_4_/NRG composite.

Figure 1 presents the overall procedure for the preparation of ZnFe_2_O_4_/NRG. When Fe(acac)_3_ and Zn(acac)_2_ were added into GO/DMF solution, the Fe^3+^ and Zn^2+^ ions can bind with the O atoms of the negatively charged oxygen-containing functional groups on GO sheets *via* an electrostatic force. During the hydrothermal process, the nitrogen was successfully doped into the network of reducing graphene. Meanwhile, ZnFe_2_O_4_ crystals can be formed and uniformly anchored on the surface of NRG, and the nano-sized ZnFe_2_O_4_/NRG can be finally obtained by the followed calcination treatment to remove residue of organic matter.

**Figure 2:**
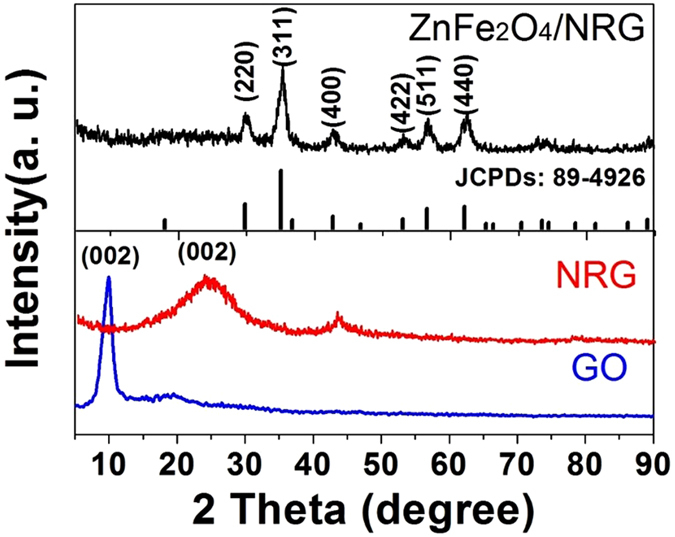
XRD patterns of GO (**a**), NRG (**b**), and ZnFe_2_O_4_/NRG (**c**) composite.

The crystallographic structure of the samples was investigated through X-ray diffraction (XRD) technique. As shown in Fig. 2, the XRD pattern of GO contains a strong and sharp peak centered at 10.5°, which corresponds to the interplanar distance (002 plane) of 0.84 nm according to Bragg equation. The larger interplanar distance of GO compared with the graphite (*d*_002_ = 0.34 nm) can be ascribed to oxidation of graphite, triggering the introduction of oxygen functional groups and the exfoliation of monolayer sheets. As contrast, a broad (002) diffraction peak of NRG can be observed at 25.3° (corresponding to the interlayer distance of 0.35 nm), indicating restacking of GO under the hydrothermal conditions. The disappearance of NRG located at 10.5° can be assigned to the thorough reduction from GO to NRG. For ZnFe_2_O_4_/NRG, the main characteristic diffraction peaks at 2θ values of 29.7°, 35.1°, 42.6°, 53.1°, 56.4° and 62.4° can be ascribed to (220), (311), (400), (422), (511) and (440) crystal planes of spinel ZnFe_2_O_4_ (JCPDS No. 89-4926), indicating a successful preparation of ZnFe_2_O_4_ without other impurity phase. The value average crystallite size of ZnFe_2_O_4_ can be calculated from the Scherrer formula: D_hkl_ = Kλ/(β cos θ), where K is a constant (0.89), θ is the diffraction angle, and β is the full width at half-maximum. The calculated crystallite size is 8.5 nm. The broad peak of RGO at 25.3° is negligible due to the strong peaks of ZnFe_2_O_4_.

**Figure 3:**
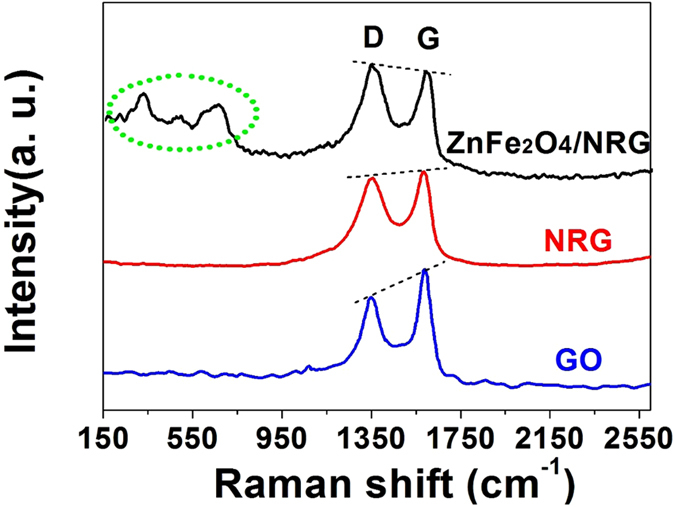
Raman spectra of GO, NRG and ZnFe_2_O_4_/NRG composite.

Raman spectroscopy has been also employed to further determine the degree of graphitization and the crystalline structure of ZnFe_2_O_4_/NRG composites. As shown in Fig. 3, two characteristic peaks of the D and G bands for these three samples are located at about 1360 and 1595 cm^−1^, respectively. It is acceptable that the intensity ratio between the D and G bands (ID/IG) can be served as a significant parameter to estimate carbon hybridization state of materials^56^,^57^. The values of ID/IG ratios for NRG and ZnFe_2_O_4_/NRG composites increase slightly from 0.80 (GO) to 0.99 and 1.02, revealing the presence of plentiful defects of NRG, which can be attributed to the heteroatomic doping of nitrogen and the firm attaching of ZnFe_2_O_4_ nanoparticles. In addition, the Raman spectrum of ZnFe_2_O_4_/NRG presents two weak peaks located at 322 and 658 cm^−1^, assigned to motions of atoms in metallic oxide, further confirming the formation of ZnFe_2_O_4_.

**Figure 4:**
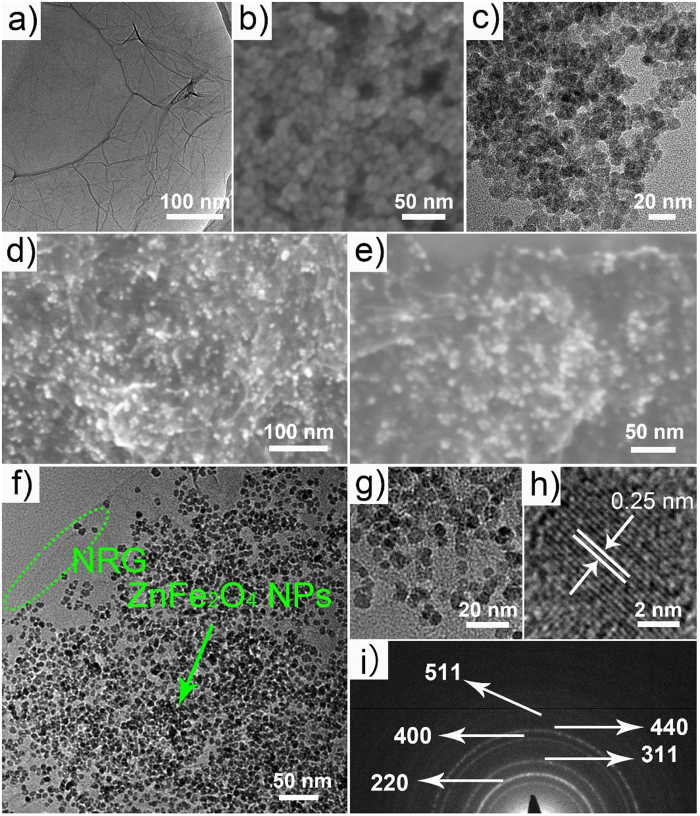
The TEM image of GO (**a**), the SEM (**b**) and TEM images of pure ZnFe_2_O_4_ NPs (**c**), Low- (**d**) and high-magnified SEM image (**e**), TEM images (**f** and **g**), HRTEM image (**h**) and SAED (**i**) of ZnFe_2_O_4_/NRG composite.

The detailed morphology and structure of the products were examined by SEM and TEM measurements, as shown in Fig. 4. The TEM image of GO (Fig. 4a) shows that the nanosheets are almost transparent as thin film and consist of a large amount wrinkles and folds, suggesting desirable properties for anchoring the ultra-small ZnFe_2_O_4_ particles. As shown in Fig. 4b and c, the pristine ZnFe_2_O_4_ nanoparticles, prepared without the addition of GO, randomly aggregate with each other with a diameter of about 9 nm. From the SEM images of ZnFe_2_O_4_/NRG (Fig. 4d and e), it can be seen that the ultra-small ZnFe_2_O_4_ nanoparticles are relatively homogeneously distributed on the wrinkled NRG at a high density. It is notably that the NRG can act as qualified support for the growth of ZnFe_2_O_4_ particles, while the decoration of ZnFe_2_O_4_ nanoparticles on NRG can effectively prevent the aggregation of the NRG. And the TGA curve of the ZnFe_2_O_4_/NRG displayed in Figure S1 revealed that the content of the rGO in the composite is about 35%. The TEM image (Fig. 4f) of ZnFe_2_O_4_/NRG can further confirm the high-dispersedly decoration of ZnFe_2_O_4_ particles on the surface of NRG. A closer observation of the ZnFe_2_O_4_/NRG (Fig. 4g) reveals that ZnFe_2_O_4_ exhibits an average diameter of 7.8 nm, which is basically consistent with the analysis of XRD, indicating the positive effect of NRG to control the nanoscale of ZnFe_2_O_4_, which is beneficial for the exposure of abundant active sites to the electrolyte. In the HRTEM image of composite (Fig. 4h), an obvious lattice between the adjacent fringes with interplanar spacing of 0.24 nm can be clearly identified, which is in agreement with the (311) plane of cubic ZnFe_2_O_4_. Besides, the selected area electronic diffraction pattern (Fig. 4i) shows some well-defined rings, which correspond to the XRD data of ZnFe_2_O_4_^58^.

**Figure 5:**
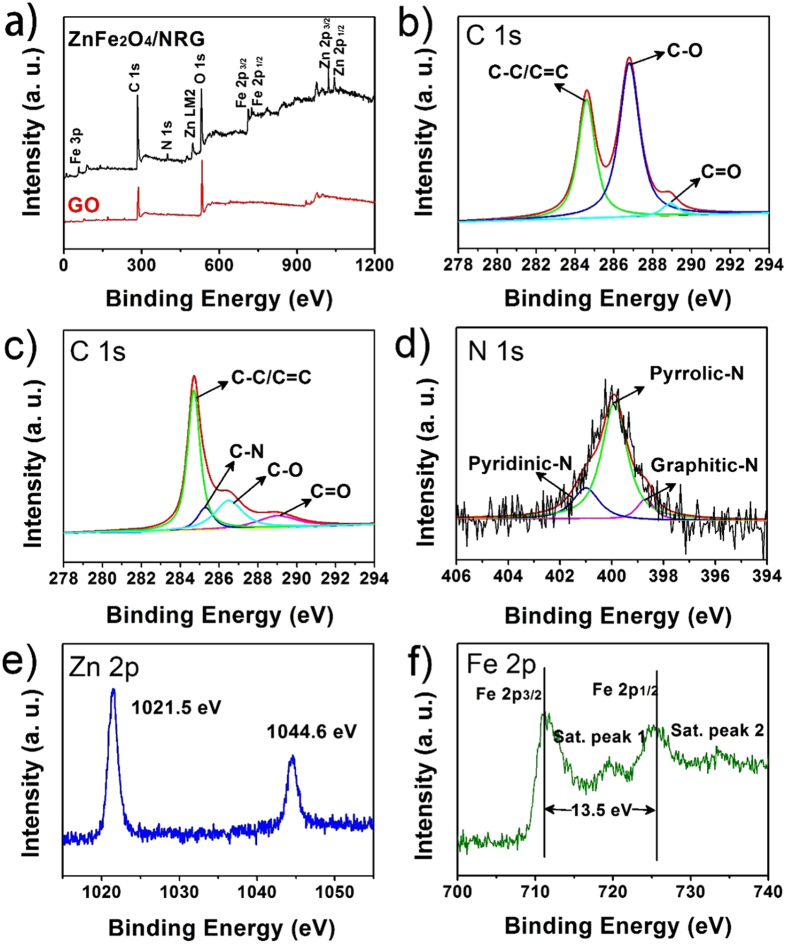
XPS survey spectra of GO and ZnFe_2_O_4_/NRG composite (**a**); C 1 s of GO (**b**) and ZnFe_2_O_4_/NRG (**c**), N 1 s (**d**), Zn 2p (**e**) and Fe 2p (**f**) of ZnFe_2_O_4_/NRG composite.

In order to clarify the chemical composition and valence of ZnFe_2_O_4_/NRG, X-ray photoelectron spectroscopy (XPS) measurements were adopted and the results are shown in Fig. 5. In contrast with GO, the existence of N, Zn and Fe in the XPS spectrum (Fig. 5a) of ZnFe_2_O_4_/NRG composite can be clearly observed. In the high resolution C 1 s spectrum of GO (Fig. 5b), there are three dominant peaks at 284.6, 286.8 and 288.8 eV, corresponding to C-C/C = C, C-O and C=O, respectively. With regard to that of ZnFe_2_O_4_/NRG (Fig. 5c), an additional peak appears at 285.3 eV, which can be associated with C-N bond. Besides, the peak intensities of C–O and C=O for ZnFe_2_O_4_/NRG are much weaker than those for the pure GO, due to the reduction of graphene oxide during the hydrothermal reaction. As shown in Fig. 5d, the N 1 s spectrum of the ZnFe_2_O_4_/NRG can be divided into three types centered at 398.7, 399.9, and 401.1 eV, assigned to Pyridinic-N, Pyrrolic-N and Graphitic-N, which can demonstrate the successful doping of nitrogen into graphene network^59^. It has been proved that pyridinic N and pyrrolic N can create large number of defects on the surface of graphene, providing more diffusion channels and active sites for the fast transportation of ions^60^,^61^. Figure 5e displayed two major peaks at 1021.5 and 1044.6 eV, which can be ascribed Zn 2p3/2 and Zn 2p1/2^62^. The high-resolution Fe 2p spectrum has been depicted in Fig. 5f. The spectrum exhibits two major peaks located at 711.2 and 724.7 eV, corresponding to Fe 2p3/2 and Fe 2p1/2, respectively, with a spin-energy separation of 13.5 eV, which is characteristic oxidation state of iron in ZnFe_2_O_4_^62^. In addition, some extra peaks donated as satellite peak around the Fe 2p3/2 and Fe 2p1/2 signals are also found. These XPS results are well consistent with the XRD and Raman analysis.

**Figure 6:**
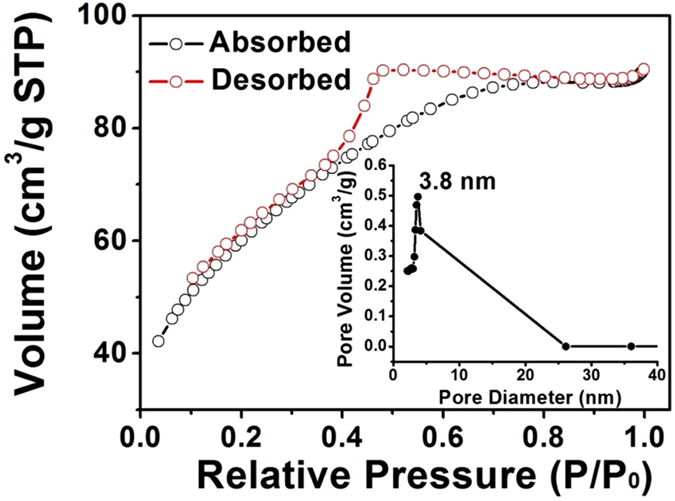
N_2_ adsorption/desorption isotherm and the corresponding pore size distribution (inset) of ZnFe_2_O_4_/NRG composite.

Surface area and pore size play important roles in determining the electrochemical properties of electrode materials^55^,^59^. Therefore, N_2_ adsorption/desorption were performed to study the specific surface area and porous nature of ZnFe_2_O_4_/NRG composites. As displayed in Fig. 6, the isotherm of ZnFe_2_O_4_/NRG possesses a typical type IV with a H3 hysteresis loop in the range between 0.4 and 0.8, manifesting the typical mesoporous structure of the composite. The Brunauer-Emmett-Teller (BET) surface area of ZnFe_2_O_4_/NRG is determined to be 212 m^2^/g. The high surface area can offer sufficient surface sites for Faradaic redox reactions, leading to better capacitive performance of the electrode materials. According to the Barrett-Joyner-Halenda (BJH) method, the pore-size distribution curve of ZnFe_2_O_4_/NRG displays an average pore diameter of 3.8 nm. This appropriate pore size can effectively favor diffusion and accession of ions into the interior voids of the materials, which contributes to high rate capability.

### Electrochemical properties

**Figure 7:**
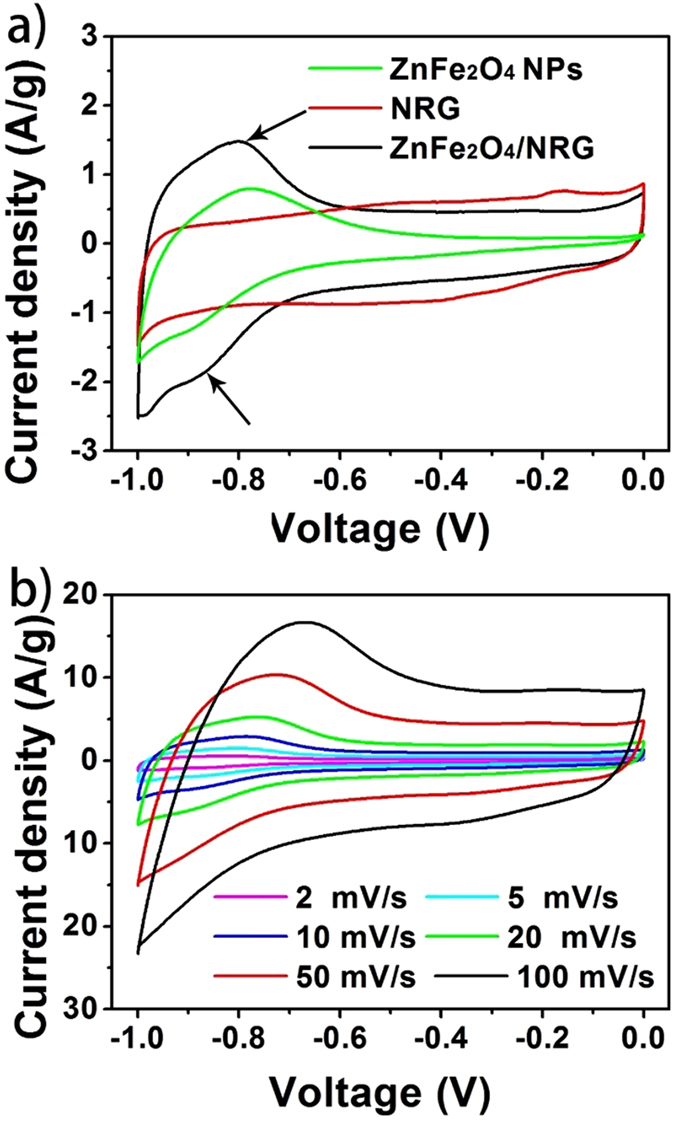
Cyclic voltammetry (CV) curves of ZnFe_2_O_4_, NRG and ZnFe_2_O_4_/NRG at a scan rate of 5 mV/s (**a**) and CV curves of ZnFe_2_O_4_/NRG electrode tested at scan rates from 2–100 mV/s (**b**).

To explore the potential of the prepared composites as supercapacitor electrodes, the electrochemical measurements have been performed in a three-electrode system using 1 M KOH as aqueous electrolyte. Figure 7a shows the cyclic voltammetry (CV) curves of ZnFe_2_O_4_ NPs, NRG and ZnFe_2_O_4_/NRG within the potential range from −1 to 0 V at a scan rate of 5 mV/s. As known, the encircled area of CV curves is proportional to the specific capacitance of the electrodes. According to this theory, we can easily deduce from Fig. 7a that the capacitance of ZnFe_2_O_4_/NRG composite is much higher than those of ZnFe_2_O_4_ NPs and NRG, due to the synesthetic effect between ZnFe_2_O_4_ and NRG. Notably, the CV curve of NRG presents a typical rectangular shape with no obvious peaks for oxidation and reduction, indicating a characteristic of the electric double layer charging mechanism. While the curves of ZnFe_2_O_4_ NPs and ZnFe_2_O_4_/NRG exhibit an anodic peak located at approximate −0.8 V and a corresponding cathodic peak potential at around −0.9 V, assigned to the reversible electrochemical reactions from ZnFe_2_O_4_ to ZnOOH and FeOOH, implying the pseudocapacitive contribution to the electrochemical performance of the electrodes^44^. The effect of scan rate on stability of the ZnFe_2_O_4_/NRG electrode was investigated with a wide scan range from 2 to 100 mV/s. As depicted in Fig. 7b, the CV curves retained similar quasi-rectangular shape at all scan rates, suggesting relatively high rate capability of ZnFe_2_O_4_/NRG, due to its unique structure beneficial for the fast ion diffusion into the electrode. Galvanostatic charging–discharging (GCD) has been also carried out to evaluate the capacitive performance of these three samples. The specific capacitance of the three electrodes can be calculated from the galvanostatic discharge curves using the following equation:


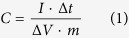


**Figure 8:**
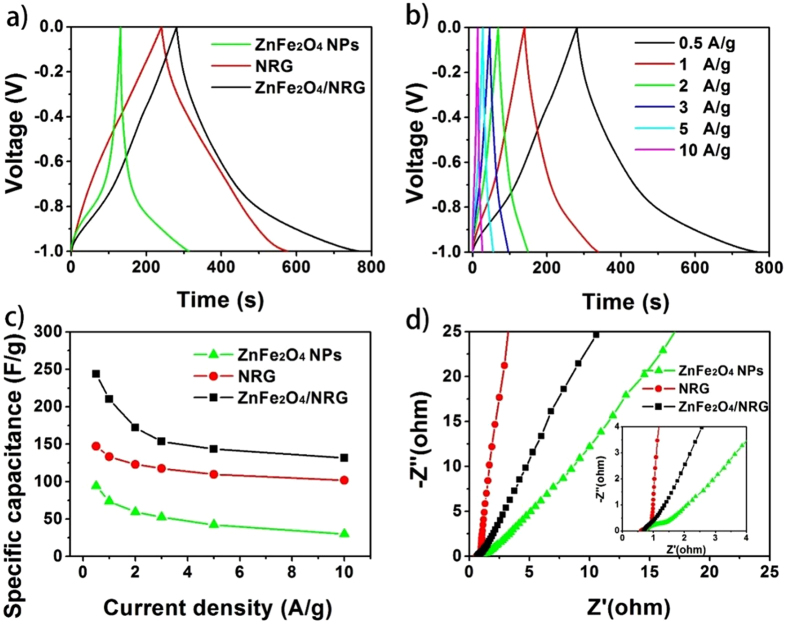
Galvanostatic charge-discharge curves of ZnFe_2_O_4_ NPs, NRG and ZnFe_2_O_4_/NRG (**a**); galvanostatic charge-discharge curves of ZnFe_2_O_4_/NRG composite tested at various discharge current (**b**); current density dependent specific capacitance of ZnFe_2_O_4_ NPs, NRG and ZnFe_2_O_4_/NRG (**c**); Nyquist plots of the EIS for ZnFe_2_O_4_ NPs, NRG and ZnFe_2_O_4_/NRG (**d**).

Where I is the response current density, Δt is the discharge time, m is the mass of the active materials on single electrode, and ΔV is the potential range during the charge-discharge measurement. Figure 8a exhibits the charge–discharge curves of the electrode materials at the current density of 0.5 A/g with voltage windows between −1 and 0 V. As expected, the ZnFe_2_O_4_/NRG electrode exhibits longest discharge time, corresponding to the highest specific capacitance of 244 F/g, which is in accordance with the analysis of CV curves.

The GCD curves of the ZnFe_2_O_4_/NRG measured at various current densities are displayed in Fig. 8b. It can be seen that the shape of the curves displays an apparent deviation from a straight line, which can be also ascribed to the feature of pseudocapacitive behavior^61^. The charge-discharge curves at all current densities can maintain a similarly symmetric shape, demonstrating high Coulombic efficiency result and low polarization from admirably reversible performance of ZnFe_2_O_4_/NRG during faradic reactions.

Good rate capability is also a key parameter to assess the potential application of supercapacitors^21^,^56^. Encouragingly, as shown in Fig. 8c, all the specific capacitances for ZnFe_2_O_4_/NRG at the same current density are much higher than those of other samples, and the specific capacitance of ZnFe_2_O_4_/NRG reached up to 244 F/g at 0.5 A/g, and retain at 131.5 F/g for a scan rate as high as 10 A/g, suggesting admirable rate performance of this composite. In contrast, for ZnFe_2_O_4_ NPs, the specific capacitance decreases rapidly from 94 F/g to 30 F/g with a very low capacitive retention rate of 32%. The possible reason for the superb electrochemical properties can be attributed to the facile combination of ZnFe_2_O_4_ NPs and NRG, which can motivate the synthetic effect between pseudocapacitors and electrical double layer capacitors. For one thing, a plenty of active sites derived from small-sized ZnFe_2_O_4_ can ensure completely faradic reaction. Moreover, the introduction of NRG can provide rapid ions transport paths and facilitate high exposure of the electroactive sites.

Electrochemical impedance spectroscopy (EIS) measurements were also conducted to obtain the electrical conductivity of each sample. As displayed in Fig. 8d, all these Nyquist plots exhibit a semicircle in the high frequency region and vertical line in the low frequency region. It is accepted that the semicircle diameter corresponds to the charge transfer impedance of the electrode and the straight line reveals the frequency dependence of ion diffusion/transport from electrolyte to the electrode surface^30^,^34^. Compared with ZnFe_2_O_4_ NPs, the ZnFe_2_O_4_/NRG owns a much smaller semicircle and a more vertical line, indicating faster reaction kinetics and lower ion diffusion resistance. The values of charge-transfer resistance for NRG, ZnFe_2_O_4_ NPs and ZnFe_2_O_4_/NRG composites are 0.59 Ω, 0.67 Ω and 0.73 Ω, respectively. The improvement of electrical conductivity can be ascribed to the indispensable role of NRG served as substrate, which can offer high contact interface for electrode and electrolyte.

**Figure 9:**
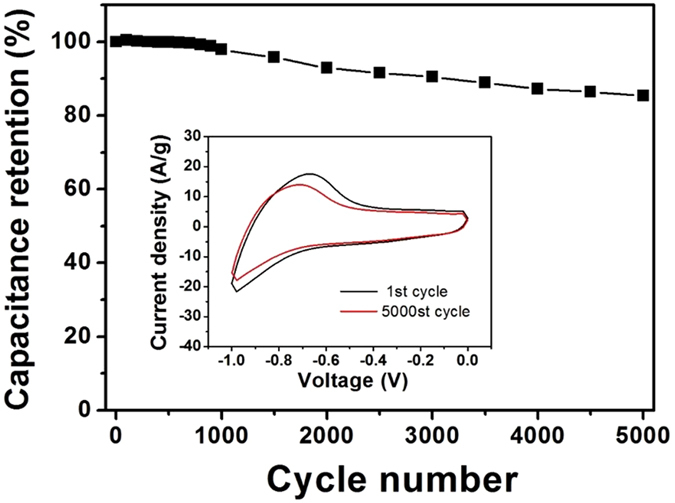
Cycling performance of ZnFe_2_O_4_/NRG composite measured at scan rate of 100 mV/s. Inset in Fig. 9 shows cyclic voltammetry curves at the first cycle and 5000th cycle.

Since long term cycling stability is a key parameter to evaluate the practical application of a supercapacitor material, ZnFe_2_O_4_/NRG electrode was also tested at 100 mV/s a potential range between −1 and 0 V for 5000 cycles, using 1 M KOH aqueous solution as electrolyte. The specific gravimetric capacitance is calculated from discharge regions of the CV using the following equation:


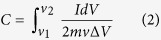


where C (F/g) is the specific capacitance of single electrode, I is the current response (mA), v_1_ and v_2_ are the vertex potentials of the voltage range, m is the grams of one electrode (g), v is the potential scan rate (mV/s), and ∆V is the voltage window (V). As shown in Fig. 9, the ZnFe_2_O_4_/NRG electrode exhibits an excellent long-term electrochemical durability, with capacitance retention of 83.8% after repetitive 5000 cycles. In addition, the CV curve (inset in Fig. 9) maintains its initial CV shape without any variation, suggesting excellent cycling stability. The excellent cycling performance of ZnFe_2_O_4_/NRG is attributable to its unique structure, which effectively improves interconnection of active materials and thus inhibits the capacitance loss during repetitive cycles.

**Figure 10:**
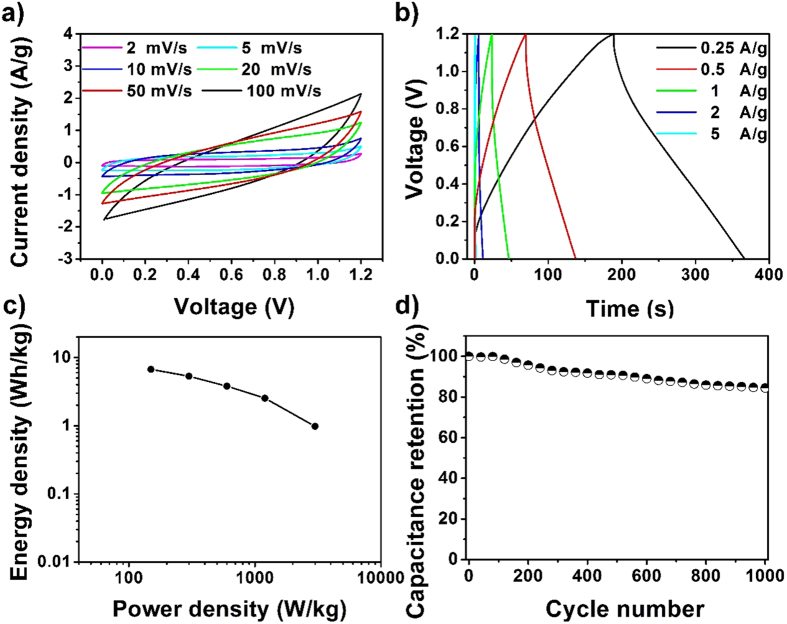
Cyclic voltammetry (CV) curves at different scan rates (**a**); galvanostatic charge-discharge curves at various current density (**b**); Ragone plot (**c**) and cycling performance at a scan rate of 100 mV/s (**d**) of the ZnFe_2_O_4_/NRG fabricated symmetric device.

To further evaluate the practical application of the ZnFe_2_O_4_/NRG composite as electrode, a symmetric supercapacitor has been designed and assembled. Figure 10a shows the CV curves of the composites at different scan rate from 2 mV/s to 100 mV/s. It can be seen that the area of the curves increases obviously with increasing potential scan rate, and the curves can maintain their shape at various scan rates, confirming the good rate behavior of the symmetric device. The galvanostatic charge/discharge curves are shown in Fig. 10b. It is noted that the charge curves are symmetrical to their corresponding discharge curves, further indicating the favorable electrochemical properties of the as-fabricated device. According to the specific capacitance of the symmetric supercapacitor at different current densities (Figure S2), the Ragone plot revealing the relationship between energy density and power density is presented in Fig. 10c. The values of energy density and power density can be calculated by the following equation:


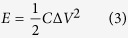



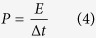


Where *E* the energy density (Wh/kg) is, *C* is the specific capacitance (F/g), Δ*V* is the cell voltage (V), *P* is the power density (W/kg), and Δ*t* is the discharge time (s). The maximum energy density and power density of 6.7 Wh/kg and 3000 W/kg can be achieved at an operating voltage of 1.2 V, which can be of potential in practical application.

In order to determine the stability of the symmetric device, CV cycling was also conducted at 100 mV/s for 1000 cycles. The capacitance of the device retention after 1000 cycles is about 84.4%, indicating an excellent long-term stability of the symmetric supercapacitor.

## Methods

### Preparation of the samples

Graphene oxide (GO) was synthesized on the basis of a modified Hummers method^63^. The prepared GO was treated with dialysis process to completely remove residual salts and acids. Certain amount of the purified GO was then dispersed and wished with DMF solution for several times. After continuous ultrasonication at room temperature for 6 h, the GO/DMF solution was obtained with a concentration about 0.8 mg/mL.

For a typical synthesis of ZnFe_2_O_4_/RNG, 0.5 mmol Zn(acac)_2_ and 1 mmol Fe(acac)_3_ were slowly added into the 40 ml of GO/DMF suspension. After stirring for 30 min, the resulting mixture was moved to a Teflon-lined autoclave followed by maintaining at 180 °C for 12 h, and then the resultant black precipitates were cleaned with ethanol and DI water several times by centrifuging. Finally, the ZnFe_2_O_4_/RNG samples were dried in a vacuum oven at 60 °C for 24 h and annealing in muffle furnace at 250 °C for 2 h to remove the residue of the organic matter. For comparison, NRG and ZnFe_2_O_4_ NPs composite were prepared in the absence of organic metal salt and GO solution under the same conditions.

### Fabrication of electrode and electrochemical measurement

The conventional three-electrode cell consisted of the counter electrode (Pt foil 1 × 1 cm^2^), the reference electrode (a Hg/HgO electrode) and working electrode (Ni foam coated with active material) employed to determine electrochemical performance of ZnFe_2_O_4_/RNG composites. The fabrication of working electrode was presented as follows. First, active material powder, acetylene black, and polytetrafluoroethylene (PTFE), with a weight ratio of 80:10:10, were mixed to form homogeneous slurry and coated on a nickel foam. After dried in a vacuum at 60 °C overnight, the obtained nickel foam was pressed under a pressure of 3 MPa to ensure firmly attachment of electrode materials. In a two-electrode system, the test capacitor was fabricated by sandwiching a porous polymer membrane separator between two as-prepared electrodes. All the tests were conducted at room temperature with a 1 M KOH aqueous solution as the electrolyte.

### Characterization

Crystalline structure, the morphology, and chemical composition of the samples were investigated by powder X-ray diffraction (XRD) (Rigaku D/_max_ TTR-III diffractometer with graphite monochromatized Cu Kα radiation (λ = 0.15405 nm)), scanning electron microscope (SEM, JSM-6480A), transmission electron microscopy (TEM, FEI Tecnai G^2^ S-Twin), high-resolution transmission electron microscopy (HRTEM), and the X-ray photoelectron spectra XPS (VG ESCALAB MK II electron energy spectrometer using Mg KR (1253.6 eV) as the X-ray excitation source). Raman spectra were conducted on a confocal laser microRaman spectrometer (LABRAM-HR, JY Co.), and N_2_ adsorption/desorption isotherms were measured from Micromeritics ASAP Tristar II 3020 apparatus. The electrochemical properties were carried out by a CHI 666D electrochemical workstation.

## Additional Information

**How to cite this article**: Li, L. *et al*. Uniformly Dispersed ZnFe_2_O_4_ Nanoparticles on Nitrogen-Modified Graphene for High-Performance Supercapacitor as Electrode. *Sci. Rep.* **7**, 43116; doi: 10.1038/srep43116 (2017).

**Publisher's note:** Springer Nature remains neutral with regard to jurisdictional claims in published maps and institutional affiliations.

## Supplementary Material

Supplementary Information
