# Supplementary material for: Uniformly Dispersed ZnFe2O4 Nanoparticles on Nitrogen-Modified Graphene for High-Performance Supercapacitor as Electrode
Source: Sci Rep. 2017 Feb 21;7:43116. doi: 10.1038/srep43116 (PMC5318878; doi:10.1038/srep43116)
Supplement: Supplementary Information [file srep43116-s1.doc]

**Supplementary Information**

**Uniformly Dispersed ZnFe_2_O_4_ Nanoparticles on Nitrogen-Modified Graphene for High-Performance Supercapacitor as Electrode**

**Lei Li,^1^** **Huiting Bi,^1^ Shili Gai,^1^ Fei He^1^, Peng Gao^1, *^, Yunlu Dai ^1^, Xitian Zhang^2, *^, Dan Yang^1^, Milin Zhang^1^ & Piaoping Yang^1, *^**


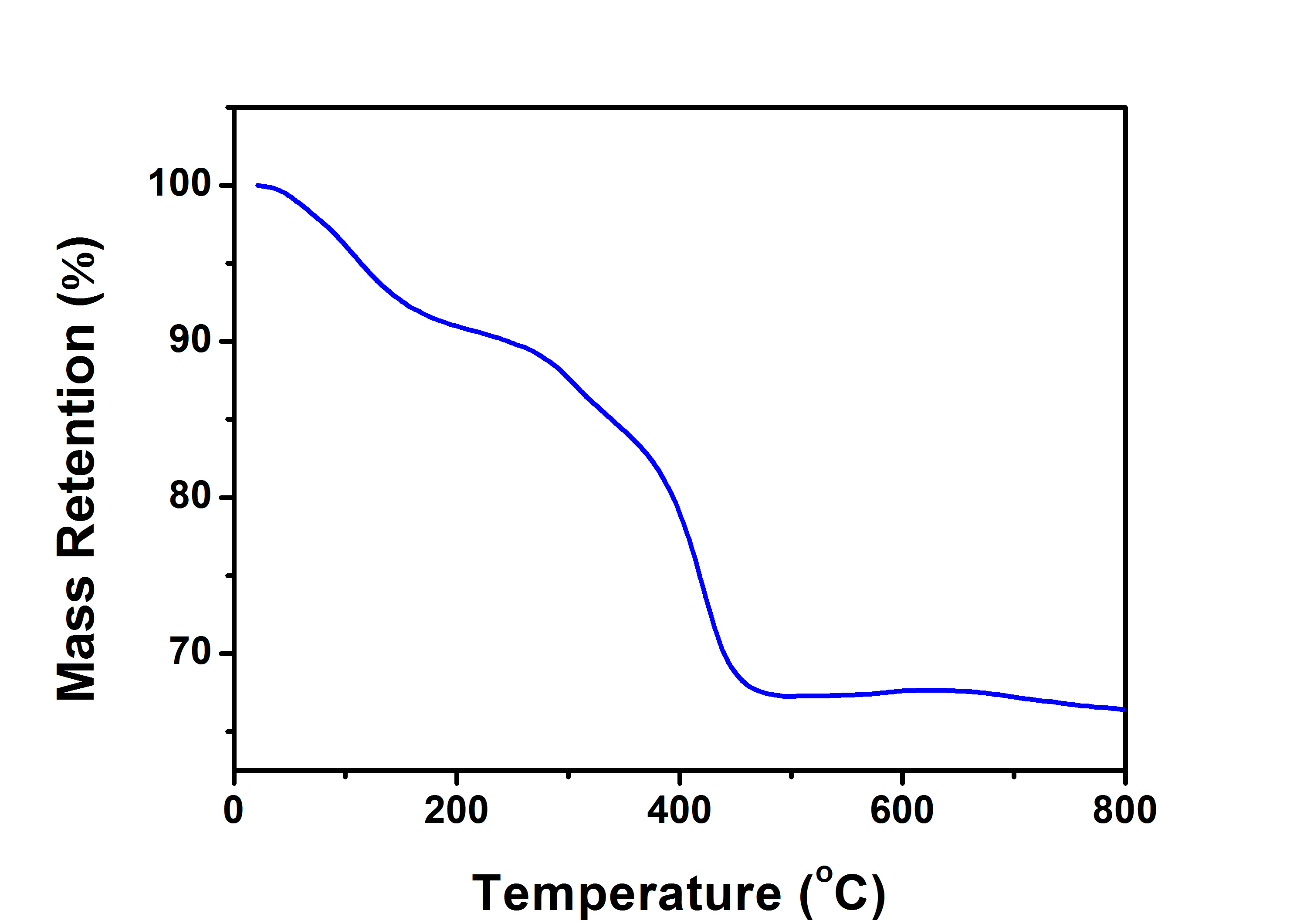


**Figure S1** TGA curve of ZnFe_2_O_4_/NRG composite

**
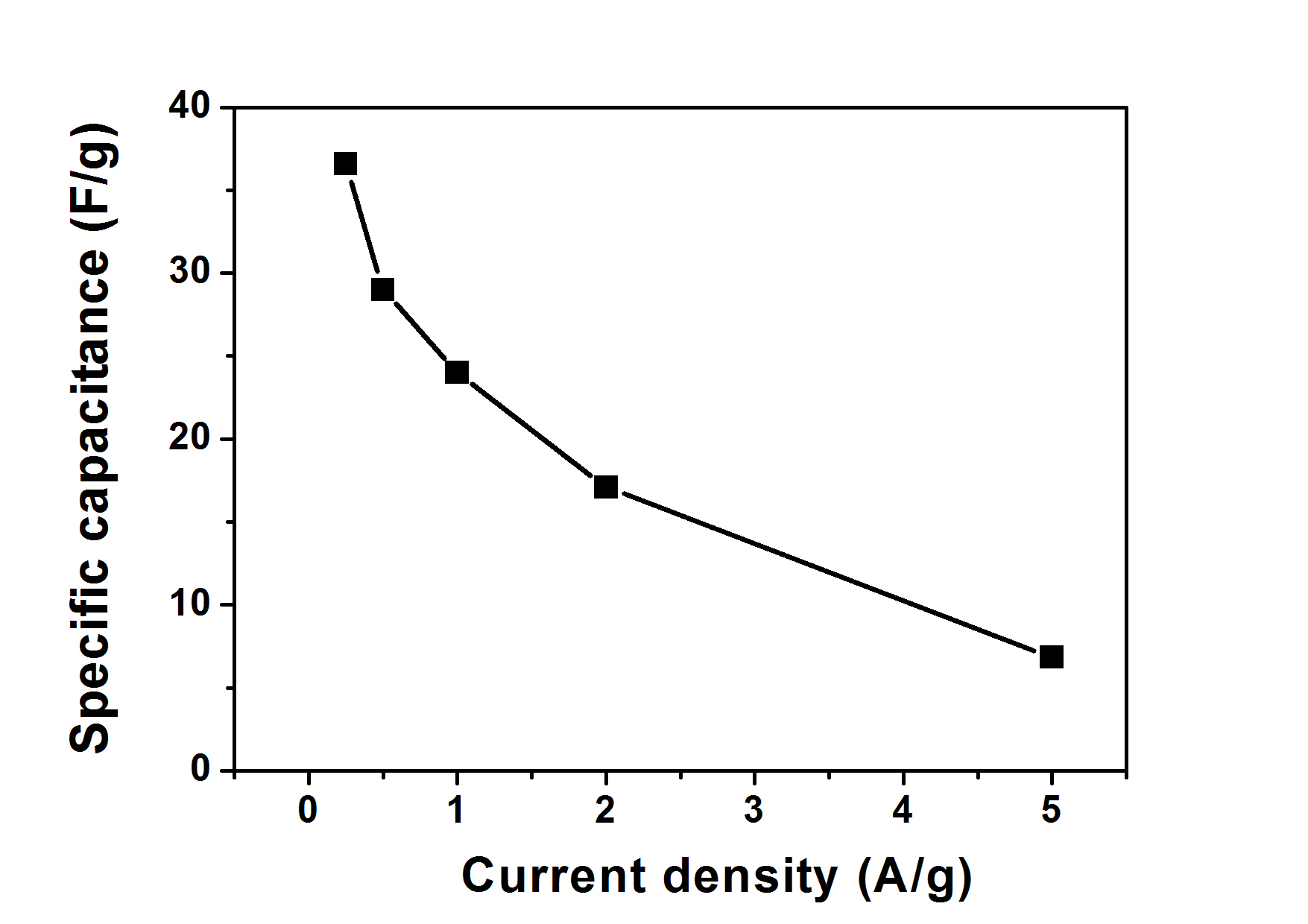

Figure S2** Current density dependent specific capacitance of the symmetric supercapacitor
